# Genomic structure of Bali cattle based on linkage disequilibrium and effective population size analyses using 50K single nucleotide polymorphisms data

**DOI:** 10.14202/vetworld.2022.449-454

**Published:** 2022-02-25

**Authors:** Pita Sudrajad, Richi Yuliavian Kusminanto, Slamet Diah Volkandari, Muhammad Cahyadi

**Affiliations:** 1 Assessment Institute for Agricultural Technology - Central Java, Indonesian Agency for Agricultural Research and Development, Ministry of Agriculture, Semarang, 50552, Indonesia; 2 Department of Animal Science, Faculty of Agriculture, Universitas Sebelas Maret, Surakarta, 57126, Indonesia; 3 Research Center for Biotechnology, Research Organization for Life Sciences, National Research and Innovation Agency (Badan Riset dan Inovasi Nasional), Cibinong, Jawa Barat, 16911, Indonesia

**Keywords:** Bali cattle, effective population size, genomic data, genetic structure, linkage disequilibrium

## Abstract

**Background and aim:**

Bali Cattle (*Bos j. javanicus*) is a local breed originating in Indonesia, accounting for 32.3% of the total cattle population. To date, no studies of the genetic structure and demographic status of Bali cattle have been conducted, even though the breeding of Bali cattle has a long and unique history that is likely to have impacted its genetic diversity. Therefore, a study that used molecular breeding technologies to characterize the demography of Bali cattle would be timely. This study aimed to examine genome diversity in Bali cattle and estimate the linkage disequilibrium (LD) and effective population size (*N*_e_) values in the cattle population.

**Materials and Methods:**

In this study, we explored the population structure and genetic diversity of Bali cattle using genomic-level analyses. Our study primarily studied cattle that had been bred in livestock breeding centers since these breeds had subsequently spread throughout Indonesia. We focused on characterizing the genetic structure, determining the level of LD present, and estimating the *N*_e_ of the Bali cattle population. The genomic data used for this study were obtained from DNA samples of 48 Bali cattle collected at the Breeding Center of Bali Cattle as well as 54 genomic samples from Bali cattle collected elsewhere in Indonesia that had been used in recent publications. This genomic dataset included exclusively 50K single nucleotide polymorphisms (SNP) array (Illumina Bovine 50SNP bead chip, Illumina, USA) data.

**Results:**

We found that the LD values of Bali cattle from the breeding center and those raised elsewhere were 0.48±0.43 and 0.39±0.40, respectively. Subsequently, the *N_e_* value of Bali cattle from the breeding center and farmers was 151 and 96, respectively.

**Conclusion:**

Our results suggest that the selection program of the breeding center is beneficial for maintaining the genetic diversity of Bali cattle.

## Introduction

Bali cattle (*Bos j. javanicus*) have a unique history and genetic background, as they are the only native Indonesian cattle that were domesticated by the Banteng [1,2]. Bali cattle are the most suitable for several regions in Indonesia because they possess unique adaptations to the tropical environment, including a high growth rate even when given low-quality feed [2]. These characteristics make Bali cattle attractive for farming in Indonesia; at the time of the last census, Bali cattle were the predominant breed in Indonesia, representing 32.3% of all cattle in the country [3]. The breeding of Bali cattle in Indonesia has a long history and began with the isolation of its ancestors on the Island of Bali. More recently, a breeding station was established, which facilitated the spreading of Bali cattle throughout Indonesia [4]. The phases of history involved likely have affected the genomes of present-day Bali cattle. Nevertheless, to date, no studies that examined the genetic structure and demographic status of Bali cattle using genomic technologies have been conducted. Decker *et al*. [5] used genome data from 20 Bali cattle in a study of the genetic composition of wild cattle from around the world, but detailed descriptions of some cattle types, including Bali cattle, remain to be explored. The use of molecular technologies in animal husbandry has become more ubiquitous, chiefly because these technologies facilitate more efficient breeding and greater genetic improvement within livestock breeding programs. At present, the emergence of new products and new findings related to genetic markers associated with desirable livestock traits present great opportunities for further improving the livestock breeding process. In Indonesia, genomic-level analyses have become an important part of research programs designed to support the improvement of livestock genetic quality. For example, such analyses have been utilized to evaluate Indonesian beef and dairy cattle genomes [4,6], as well as the relationship between *PLAG1* (a gene on chromosome 14 of the cattle genome) with Ongole cattle birth weight [7].

Single nucleotide polymorphisms (SNPs) are a type of variation in nucleotide sequence found in DNA. SNPs are often stable and may also be directly related to protein function; therefore, they are one of the best genetic markers used by breeding programs [8]. A project to explore sequence variation in the cattle genome began in 2003 and was published in 2004, revealing that several SNPs were found [9]. Currently, automated SNP detection devices exist that can search for SNP variation in the cattle genome; these include the Bovine SNP50 Beadchip v3, which can detect up to 53,218 SNPs in the cattle genome (Illumina Inc., USA).

Linkage disequilibrium (LD) is one of the important parameters in population genetics; it reflects the degree of correlation between allelic variants found at different loci in a given population. Therefore, LD values reflect the distance between genetic markers and the level of inbreeding (genetic proximity) of individuals in a population. LD values are smaller if the genetic distance is greater; this may reflect historical migration, mutation, recombination, or selection in a population [10,11]. LD values can also be used to estimate population genetic parameters such as the effective population size (*N_e_*) [4,12]. Understanding the *N_e_* is critically important for trying to maintain genetic diversity in a livestock population, since a high *N_e_* reflects a great deal of genetic variation within this population. In contrast, a low *N_e_* indicates that less genetic variation is present.

This study aimed to examine genome diversity in Bali cattle and estimate the LD and *N_e_* values in the cattle population. Specifically, we seek to examine the genomic effects of the selection and breeding programs of the Breeding Center of Bali Cattle Breeding; to do so, we will compare LD and *N_e_* values of cattle bred at the breeding center with other Bali cattle populations bred by private farmers. Estimates will be obtained using the Bovine 50SNP Beadchip (Illumina Inc.). The results of this study may be useful as an assessment of breed development in Indonesian Bali cattle and may also be used as a basis for future policies regarding Bali cattle.

## Materials and Methods

### Ethical approval

All animal procedures related to sample collection were approved by the Ethical Clearance Commission at National Research and Innovation Agency (Badan Riset dan Inovasi Nasional) No. 82/Klirens/X/2021.

### Study period and location

The study was carried out from March to November 2021. A total of 48 Bali cattle blood samples were obtained from the Breeding Center of Bali Cattle (Denpasar, Indonesia). The DNA isolation and related works were conducted at the Division of Biology, Integrated Laboratory of Universitas Sebelas Maret. In addition, genomic analysis was conducted at Macrogen (Korea).

### Genomic data sources for Bali cattle

We obtained genomic data from 102 samples of Bali cattle. Forty-eight of these samples came from DNA isolated from the blood of 48 Bali cattle bred at the Breeding Center of Bali Cattle (Denpasar, Indonesia). The other 54 samples included 20 samples uploaded by Decker *et al*. [5] to the DRYAD database (http://datadryad.org/), 18 samples from the appendix of a publication by Hartati *et al*. [7], and 16 samples sourced from data of Sudrajad [13], which we used to represent Bali cattle bred by farmers. The Bali cattle at the breeding center were produced by a series of quantitative and qualitative selection processes designed to breed superior cattle [14]. By contrast, Bali cattle produced by farmers are simply those that had been bred according to the habits of the farmers that owned them.

DNA isolation was carried out as per the method of Sambrook *et al*. [15] at the Integrated Laboratory of Universitas Sebelas Maret (Surakarta, Indonesia). DNA concentration quantification was performed using Picogreen (Thermo Fisher Scientific Inc., USA), and evaluations of DNA purity were performed using a NanoDrop (Thermo Fisher Scientific Inc., USA) device. DNA samples were deemed to be of sufficient quality if the concentration was at least 20 ng/μL and the absorbance ratio at 260 and 280 nm was more than 1.8. DNA samples were then screened using the Illumina Bovine SNP50 v3 Beadchip (Illumina Inc.) at Macrogen (Korea) to obtain genomic data. This chip contains a genotyping array comprising 53,218 SNPs spread uniformly throughout the bovine genome.

### Genome data quality control

Before analyzing the genomic structure and genetic diversity of Bali cattle, we filtered our genomic data to remove low-quality data using PLINK v1.07 (Purcell Lab, Harvard Medical School, Boston, USA) [16]. Quality control filtering for genotype data was performed using the following criteria: SNP variants would be maintained if the Hardy–Weinberg Equilibrium value was not <1×10^−4^, the SNP call rate was >90%, the minor allele frequency was <1%, and the proportion of empty SNP variants for each individual and the proportion of empty genotypes for each variant must not exceed 10% [13]. In addition, our dataset included all autosomes, which in cattle includes chromosomes 1-29.

### Analysis of the genetic structure

Analysis of the genetic structure was performed in both Bali cattle populations. First, we calculated the expected heterozygosity (*H_e_*) and observed heterozygosity (*H_o_*) values and determined the value of the inbreeding coefficient (*F_IS_*) as well as the average genomic relationship matrix (GRM). The *H_o_* and *H_e_* values were calculated using the *hierfstat* package as implemented in R v.3.2.2 (The R Foundation for Statistical Computing, Vienna, Austria) [17]. These calculations are based on the formula compiled by Nei [18], as follows:


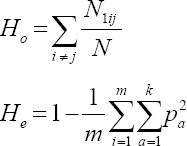


*N*: The number of samples tested

*N*_1*ij*_: Heterozygosity at each locus

*m*: The number of loci tested

p^2^_a_:Allele frequency – a from all k allele

The *F_IS_* value was also calculated using the same R package (The R Foundation for Statistical Computing) through the formula described by Weir and Cockerham [19], as follows:


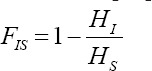


*H_I_*: The average *H_o_* of all samples in a population

*H_S_*: The average *H_e_* of all samples in a population

The GRM value was calculated using Genome-wide Complex Trait Analysis v1.25.2 (University of Queensland, Australia) [20]. This program generates two outputs: The first corresponds to SNP relationships within an individual (diagonal), and the second corresponds to SNP relationships between individuals in the same population (off-diagonal). Sample variability within each population was determined using a negative value on the off-diagonal variance.

### LD

Various statistics have been introduced to measure LD. In our study, we calculated the LD using the *r*^2^ formula [21], as follows:


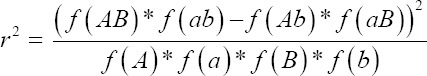


*f(x)* designates the frequency of the *x* allele. LD value estimation was performed using PLINK v1.07 (Purcell Lab) [16] and was visualized using the size of the allele distance (kb) through R v.3.2.2 (The R Foundation for Statistical Computing) [17]. PLINK was used as a measurement tool, and the -*-r2* command was used to obtain the LD value of SNP pairs. Next, the -*-ld-window-r2* command was used to report all SNP pairs [16]. LD values range from 0 to 1; a value of 1 indicates a strong correlation between variants.

### Effective population size (*N_e_*)

The effective population size (*N_e_*) was estimated using the LD value and Sved’s formula, as presented by Xu *et al*. [12], as follows:


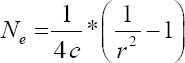


*c* designates the recombination distance in Morgan’s units. *N_e_* was estimated using R v.3.2.2 (The R Foundation for Statistical Computing) [17]. Next, *N_e_* was plotted following estimated times in horizontal ordinate, which was obtained by (*2c*)^–1^. We graphed the resulting relationship using the *ggplot2* package in R to obtain usable plots.

## Results and Discussion

### Bali cattle genome data conditions

**Supplementary Figure-1:**
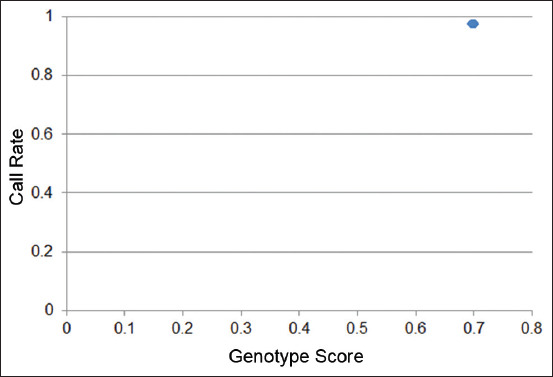
The level of reliability of SNPs in the Bali cattle genome.

The SNP microarray we utilized in this study screened for 53,218 SNPs (Illumina Inc.). Our genotyping dataset showed that the average percentage of SNPs detected (i.e., the call rate) in the Bali cattle genome was 97.8%. In addition, the average genotype score for each SNP was 0.7. These figures suggest that the level of reliability of the SNP data used in this study is high. Supplementary Figure-1 presents a further analysis of the reliability of our genotyping dataset.

**Figure-1:**
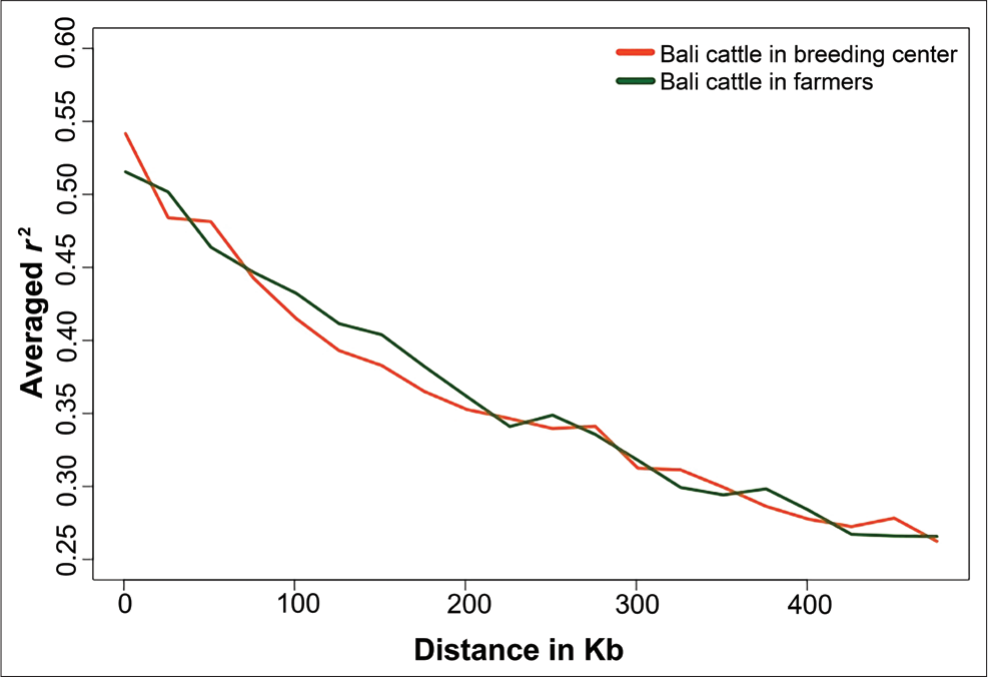
Graph of LD value of Bali cattle populations.

The number of SNPs from Bali cattle genome data from the breeding center obtained after the genome data quality control process was 49,439 variants (93%). Conversely, the number of clean SNPs in farmers’ Bali cattle genome data was 52,886 variants [13]. The reduced number of variants is mainly because some genotypes cannot be well identified during the genotyping process [16].

### Structure of the Bali cattle genome

Table-1 [5,7,13] summarizes the results of our comparative genome structure analysis of the two types of Bali cattle. Table-1 shows that the Bali cattle samples from the breeding center showed higher heterozygosity than the samples obtained from the farmers’ Bali cattle. In addition, the inbreeding coefficient and the measure of relationships between individuals from the breeding center population samples were also lower than the corresponding measurements of the samples of the farmers’ cattle. At the same time, the effective population size value was much higher. Taken together, these figures indicate that Bali cattle from the breeding center are more diverse and have lower levels of inbreeding than the farmers’ cattle.

**Table 1:** Summary statistics of observed Bali cattle populations.

Bali cattle population	No. of samples	Observed SNPs in BTA	*H_o_* ^1^	*H_e_* ^2^	*F*_IS_^3^	GRM^4^		LD^5^ (SD)	Recent *N_e_*^6^

						Diagonal	Off-diagonal		
Breeding center	48	49,439	0.30	0.26	−0.19	0.70	−0.021	0.48 (0.43)	151
Farmers^7^	54	52,886	0.12	0.08	−0.16	0.57	−0.011	0.39 (0.40)	96

1 Observed heterozygosity.

2 Expected heterozygosity.

3 Inbreeding coefficient.

4 Average of the genomic relationship matrix referring to inbreeding (diagonal) and outbreeding (off-diagonal).

5 Linkage disequilibrium as estimated using the *r*^2^ method.

6 Effective population size.

7 Data sources: Decker *et al*. [5], Hartati *et al*. [7], and Sudrajad [13]

Furthermore, the diagonal GRM value (which represents the average relationship between variants in an individual within a population) of the Bali cattle from the breeding center was higher than that of Bali cattle reared by local farmers (Table-1). This indicates that the variant in the Bali cattle from the breeding center is uniformly present in that population. This is likely a result of the cattle breeding program applied by the breeding center to select desirable characteristics. When considered together, our results suggest that the selection program of the Bali cattle breeding center is successful. Moreover, our findings agree with other genomic structure analyses of livestock populations [22].

### LD

The uniformity of variants present in the genomes of Bali cattle from the breeding center can be verified by checking the average LD value. As per our knowledge, this the first study of LD in Bali cattle; our results show that the calculated LD value of the Bali cattle population from the breeding center (0.48±0.43) was higher than the LD value of Bali cattle bred by farmers (0.39±0.40) (Table-1). This indicates that LD patterns will be population-specific, since LD depends on the genetic events experienced by individuals within a population [23]. We hypothesize that the higher LD of Bali cattle from the breeding center might be caused by the selective breeding conducted on the population, especially since other farmer-bred Bali cattle did not experience a similar selection. This is significant because selection can affect the level of variance uniformity [24]. Moreover, since high LD values result from recent selection, low levels of LD may, therefore, reflect a low selection intensity in the farmer-bred population [25,26].

Figure-1 shows a graph of changes in the estimated LD values in interallelic distances up to 500 kb for Bali cattle from the breeding center. Here, we see that average LD values were high over a short allele distance and continually decreased as the allele distance increased. This trend has also been found in other cattle populations [11,12,23,26]. Moreover, when compared with LD values of cattle from other countries as calculated by Perez O’Brien *et al*. [26], the LD values of the Bali cattle population bred at the breeding center were higher than the corresponding values for *Bos indicus* cattle (0.39) but lower than for *Bos taurus* cattle (0.59). By contrast, Bali cattle bred by farmers showed average LD values that were equivalent to those of the *B. indicus* cattle. In general, LD values can differ between populations even within a single cattle breed, since large LD values are dependent on changes in genetic composition experienced by individuals in the population [11,23]. LD values will tend to be low if the selection intensity in the population is low, and there is the possibility of crossing with native animals, as is often the case with *B. indicus* cattle in developing countries [26].

### Effective population size (*N_e_*)

**Figure-2:**
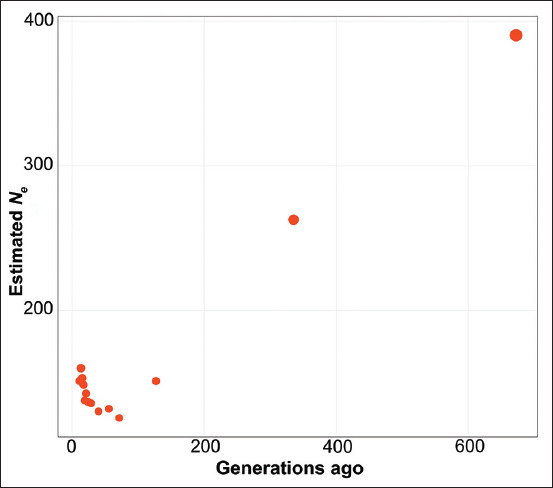
Graph of the effective population size of Bali cattle.

In this study, we found strong trends for *N_e_* values in the Bali cattle population. Relative to *N_e_* values from several generations ago, the effective population size of Bali cattle has experienced a sharp decline. However, in the more distant past (i.e., from 40 generations ago until the present) *N_e_* values were increased (Figure-2; Supplementary Table-1) [5,7,13]. This is in contrast to the most common pattern, in which the *N_e_* of a population was much higher in the past and has decreased continuously until the current generation [4,12,23,25].

The effective population size illustrates a pattern of genetic variation that can be used to explain population diversity from the past to the present [23]. Thus, we constructed a graph of the *N_e_* values of the Bali cattle populations in this study. This graph illustrates that there have been efforts to increase the genetic variation within the Bali cattle population since approximately 40 generations ago. Taken together, our analysis of effective population size shows that in Indonesia, the establishment of a Bali cattle breeding unit in 1976 and the subsequent distribution of Bali cattle to other regions, as well as efforts to artificially breed Bali cattle [2,4,14,22], have helped to maintain genetic diversity in the studied populations of Bali cattle.

The *N_e_* values of the two Bali cattle populations studied here are both far greater than the minimum threshold of the Food and Agricultural Organization for determining whether a cattle population is far from extinction (i.e., an effective population size of 50 for each generation) [27]. Future efforts should then help to maintain the genetic diversity of Bali cattle populations.

## Conclusion

Based on observed heterozygosity levels, inbreeding coefficients, and GRM values, we conclude that the Bali cattle population bred at the breeding center has a higher level of genetic diversity than the Bali cattle bred by farmers. Moreover, the LD value of Bali cattle falls just above *B. indicus* and just below *B. taurus*, and the effective population size remains high. The livestock selection program implemented by the Breeding Center of Bali Cattle may, therefore, be influential in helping to maintain the genetic diversity of Bali cattle. The results of this study can be used as a basis for developing and improving Bali cattle breeding policies in the future.

## Authors’ Contributions

PS and MC: Conceptualization, investigation and data curation, software, validation, writing original draft and review and editing. RYK: Investigation and data curation and Methodology. SDV: Investigation and data curation, methodology, validation, and review and editing. All authors read and approved the final manuscript.

## Supplementary materials

**Supplementary Table 1:** The historical effective population size (*N_e_*).

Bali cattle in the breeding center																									
*N_e_*	151	160	153	148	137	142	136	135	130	132	125	151	262	390	766	1980	-	-	-	-	-	-	-	-	-
Generation	11	13	15	17	19	21	23	28	40	55	71	127	335	672	1482	5104	-	-	-	-	-	-	-	-	-
**Bali cattle in the locals^1^**																									
*N_e_*	96	112	118	119	133	128	136	143	155	170	173	193	191	230	269	305	324	377	528	515	613	737	773	769	-
Generation	12	14	16	19	22	26	31	37	44	53	65	79	97	120	149	186	233	292	367	452	553	658	756	848	-

1 Data Sources: Decker *et al*. [5], Hartati *et al*. [7], Sudrajad [13]
